# A study of Clinical Profile and in Hospital Outcomes of patients undergoing Percutaneous Transvenous Mitral Commissurotomy at a Tertiary Care Center of Nepal

**DOI:** 10.1016/j.amsu.2022.104867

**Published:** 2022-11-15

**Authors:** Sutap Yadav, Sangam Shah, Ratna Mani Gajurel, Chandra Mani Poudel, Roshan Ghimire, Nischal Shah

**Affiliations:** a Department of Cardiology, Manmohan Cardiothoracic Vascular and Transplant Center, Maharajgunj, Nepal; b Tribhuvan University, Institute of Medicine, Maharajgunj, 44600, Nepal

**Keywords:** PTMC, Mitral stenosis, Outcomes, Mitral regurgitation, Mitral valve area, Nepal, PTMC, percutaneous transvenous mitral commissurotomy, AF, atrial fibrillation, BMV, balloon mitral valvotomy, CMC, closed mitral commissurotomy, LA, left atrium, LAP, left atrial pressure, MS, mitral stenosis, MVA, mitral valve area, MVR, mitral valve replacement, NSR, normal sinus rhythm, NYHA, New York Heart Association, OMC, open mitral commissurotomy, RHD, rheumatic heart disease, PA, pulmonary artery, PASP, pulmonary artery systolic pressure, TTE TEE, Transthoracic Echocardiography Transesophageal Echo, LVEF, Left ventricular ejection fraction, LVSD, Left ventricle systolic dysfunction, MI, Myocardial Infarction

## Abstract

**Introduction:**

Rheumatic heart disease (RHD), is a common cause of mitral stenosis (MS) in developing nations. As per current recommendation, Percutaneous Transvenous Mitral Commissurotomy (PTMC) is advised as a Class IA (I-Class Of Recommendation, COR; A-Level Of Evidence, LOE) indication in patients with symptomatic severe mitral stenosis. We aim to examine the clinical profile and in-hospital results of PTMC for mitral stenosis.

**Methods:**

A cross-sectional retrospective study was conducted at Manmohan Cardiothoracic Vascular and Transplant Center from April 2020 to May 2022. A structured questionnaire was used to collect the data and ethical approval for conducting the study was taken from the Institutional Review Committee (IRC) of Institute of Medicine (IOM). The data was collected in Microsoft Excel (Ver. 2013). For statistical analysis, SPSS 21 (IBM Corp. Released 2012. IBM SPSS Statistics for Windows, Version 21.0. Armonk, NY: IBM Corp.) Association was measured using a parametric and non-parametric test (depending upon the distribution of data) and p value < 0.05 was considered significant.

**Results:**

A total of 104 patients who met the inclusion criteria underwent PTMC during the study period. The mean age group of the patient was 41.7 ± 12.5 years, of which 23 (22.1%) were males and 81 (78.9%) were females. Mean mitral valve area prior to PTMC was 0.98 ± 0.19 mm^2^ that increased to 1.69 ± 0.19 mm^2^ after the procedure and it was statistically significant (p=<0.001). The post PTMC MVA varied with PTMC Wilkin's score with less than or equal to 8 having favorable outcomes.

**Conclusion:**

Successful PTMC is highly influenced by the patients' increasing age, valve morphology (calcification, thickness, mobility), Left atrial dimensions, Pre PTMC mitral valve area, Degree of Baseline mitral regurgitation. Post procedure development of MR is usually well tolerated but rarely be severe enough requiring surgical valve replacement.

## Highlights

• Rheumatic heart disease (RHD), is a common cause of mitral stenosis (MS) in developing nations.
• Mean mitral valve area prior to PTMC was 0.98 ± 0.19 mm^2^ that increased to 1.69 ± 0.19 mm^2^ after the procedure.
• Successful PTMC is highly influenced by the patients' increasing age, valve morphology left atrial dimensions, pre PTMC mitral valve area, degree of Baseline mitral regurgitation.

## 1 Introduction

The prevalence of rheumatic heart disease (RHD), which is a common cause of mitral stenosis (MS) in developing nations, was reported to be 72.47% in Nepal [1]. The closed mitral commissurotomy (CMC), a surgical procedure for treating severe mitral stenosis, was first established in the 1940s [2]. Open surgical mitral commissurotomy (OMC) and replacement of the mitral valve became the preferred surgical procedures with the advent of cardiopulmonary bypass in the 1960s. After the emergence of percutaneous balloon dilatation procedures, as described by Inoue in 1984 and Lock et al., in 1985, the percutaneous approach became the treatment of choice for appropriate valves [3,4]. Further modifications to their methods have produced better outcomes.

Its effectiveness in treating children and young adults using the Inoue balloon has previously been proven. Growing interest in this technique has led to its wider applicability to circumstances where surgical commissurotomy would not typically have been contemplated, such as in elderly patients with calcific MS [5,6]. In a Comprehensive Valve Center, as a Class IA recommendation, PTMC is indicated in symptomatic patients (NYHA classes II, III, or IV) with severe rheumatic MS (mitral valve area ≤1.5 cm^2^, Stage D), favorable valve morphology and less than moderate (2+) MR without left atrial (LA) thrombus [7]. Because the mechanism of valve dilation closely resembles the mechanism of surgical mitral commissurotomy, PTMC has grown in popularity and is now regarded as the preferred course of treatment for patients with symptomatic severe Mitral stenosis and good valve morphology. It's important to highlight that the post-procedural increase in mitral valve area is primarily caused by commissural splitting. The objective of our research is to examine the clinical profile and in-hospital results of PTMC for mitral stenosis.

## 2 Methods

### 2.1 Study design and setting

This study was conducted retrospectively in Manmohan Cardiothoracic Vascular and Transplant Center, located in Maharajgunj, Kathmandu. This center was chosen for study because of its high flow of cardiac patients and patient from all Nepal are referred here as it is tertiary center. Ethical approval for conducting the study was taken from the Institutional Review Committee (IRC) of Institute of Medicine (IOM). [Approval number: 398 (6-11) E^2^ 078/079] This case has been reported as per STROCSS 2020 criteria [8].

### 2.2 Study participants and eligibility criteria

All the patients meeting the inclusion criteria admitted in Cardiology department from April 2020 to May 2022 were selected for the study. To highlight, the number of elective cases being done were fewer than usual due to the COVID Pandemic.

The inclusion criterias were consecutive patients of age more than 18 yrs, symptomatic severe mitral stenosis with favorable valve morphology, previous successful PTMC or surgical commissurotomy, and those who provide written consent for the study. Patients with Mitral valve area≥1.5 cm^2^, Patients with moderate to severe mitral regurgitation grade >2/4, Infective Endocarditis, presence of left atrial thrombi assessed by Transesophageal Echocardiography (TEE), severe aortic valve disease, severe tricuspid valve disease, severe bi-commissural calcification, and coronary artery disease (CAD) requiring surgery, other valves involvement requiring surgical intervention, coronary artery bypass grafting patients who had limited life expectancy and critically ill were patients were excluded from the study.

### 2.3 Sampling

We included all the patients with complete data and who had undergone PTMC. Hence, sampling was not done.

### 2.4 Study tools and techniques

A structured questionnaire was used to collect the data retrospectively from the admitted patients. Written informed consent was taken from the hospital record for collection of data.

### 2.5 Study variables

The variables were categorized under socio-demographic factors, personal factors, and other study variables. The age and sex of the patient were included under sociodemographic factors. Similarly, patients’ comorbidities, smoking, and alcohol consumption habits were recorded under personal characteristics.

## 3 Procedure

In the absence of more than mild mitral regurgitation, successful PTMC was defined as a reduction in mean left pressure of more than 50% from baseline or a decrease in *trans*-mitral gradient of half the initial value, an increase in MVA of more than 50% from baseline, and a valve area of more than 1.5 cm^2^ and without a >2+ rise in mitral regurgitation severity. Any increase in mitral valve area less than that described as success was considered suboptimal PTMC. Inability to cross/dilate the mitral valve was characterized as procedural failure. Mean LA pressure and mean mitral valve gradient before and after PTMC were procedure-related variables.

All of the patients underwent a thorough clinical and echocardiographic 2D-ECHO, Doppler, and color flow imaging examination evaluation to assess the severity of MS, valve morphology, and mitral regurgitation prior to PTMC and within 24 h after procedure. To rule out the presence of a LA clot, a transesophageal ECHO was performed a day before or on the same day prior to the procedure. In patients receiving anticoagulation, INR values were checked prior to PTMC. To reduce the risk of bleeding, patient underwent the procedure only if their PT/INR was less than 1.5.

Under local anesthesia, PTMC was performed under aseptic conditions using the right femoral venous approach. LA pressure was measured before and after the balloon inflation. The following formula was used to determine a simple balloon (Inoue) sizing procedure based on body height for selecting a suitable sized balloon catheter.

Balloon size = [Height (in cm)/10] + 10

After cleaning and draping, the appropriately sized sheaths are inserted into Right Femoral Vein (RFV) and Right Femoral Artery (RFA). Under fluoroscopic guidance, *Pigtail catheter* is inserted via RFA sheath and is deployed in the ascending aorta. Under the guidance of terumo wire, *Mullin sheath* is inserted via Right Femoral Vein sheath and advanced to the Right atrium. A puncture is made in the interatrial septum with the help of the *Septal puncture needle*. After performing a transseptal puncture at the mid fossa ovalis, heparin is given with the goal of achieving an active clotting time (ACT) of 250 s or less.

The LA pressure and left ventricular (LV) pressure are then monitored simultaneously to determine the *trans*-mitral gradient. *Coil wire* is then passed through the interatrial septal puncture to the left atrium. Then, dilatation of the punctured interatrial septum is done with the help of the dilator. *Inoue Balloon* is then passed through the dilated IAS to LA; is negotiated through the mitral valve to LV cavity with the help of the *J-wire* and is then inflated with contrast mixed water solution. With the distal balloon only partially inflated and the stylet removed by 3–5 cm, the balloon will advance and cross the mitral valve (MV). The stylet can be rotated counterclockwise to help guide the balloon across the MV. After being fully inflated, the distal balloon is repeatedly moved back and forth to make sure it is not ensnared in the chordae. After that, it is gently pressed against the MV as the proximal balloon is swiftly inflated to accomplish valvuloplasty. The balloon is then dragged back into LA after being deflated. Transthoracic Echocardiography/Transesophageal Echo (TTE/TEE) is used to evaluate the effectiveness of valvuloplasty, specifically the level of stenosis that remains and the severity of MR. The procedure is repeated at 1 mm below the first level if there is still considerable stenosis with mild to moderate MR. valvotomy done. Finally, sheaths are removed compression applied over the right inguinal area.

Pre and post procedure echocardiographic evaluation were done. Early in diastole, Mitral Valve area as obtained using 2D planimetry in a parasternal short axis view. In end systole, just prior to mitral valve opening, the left atrial size was quantified in a parasternal long axis view on a 2D echocardiography. Mitral regurgitation was graded 1+ to 4+ using a color doppler in an apical 4 chamber view, and the amount of regurgitant jet.

### 3.1 Statistical analysis

Data was compiled, edited, and checked daily to maintain consistency. The data was collected in Microsoft Excel (Ver. 2013). For statistical analysis, SPSS 21 (IBM Corp. Released 2012. IBM SPSS Statistics for Windows, Version 21.0. Armonk, NY: IBM Corp.) was used. Descriptive analysis was done to identify the distribution of socio-demographic characteristics of patients, and the association was measured using a parametric and non-parametric test (depending upon the distribution of data). A p-value <0.05 for the two-tailed test was considered statistically significant.

## 4 Results

We included 104 patients who underwent PTMC. The mean age group of the patient was 41.7 ± 12.5 years, of which 23 (22.1%) were males and 81 (78.9%) were females. Of the included females 5 (4.8%) of them were pregnant. Majority of the patients were in were in NYHA class III (62.5%). Similarly, 14 (13.5%) were in NYHA class II while 23 (22.1%) belong to NYHA class IV. Previous PTMC and CMC candidates 11(10.6%) and 5 (4.8%) were also included. Systolic LV dysfunction was present in 12 (11.53%) of the patients. Sinus rhythm was present in 69 (65.7%) patients while arrhythmias in the form of atrial fibrillation was found in 31 (29.5%) patients. The details of the demographic variables are shown in Table 1.

**Table 1:** Demographic profile and baseline characteristics of the patients.

Mean age (years)	41.7 ± 12.5	P value
**Gender**		
Male	23 (22.1%)	
Female	81 (78.9%)	
**NYHA Class**		
I	2 (1.9%)	
II	14 (13.5%)	
III	65 (62.5%)	
IV	23 (22.1%)	
**ECG**		
Sinus rhythm	69 (65.7%)	
Atrial Fibrillation	31 (29.5%)	
2:1 Atrial flutter	3 (2.9%)	
4:1 Atrial flutter	1 (1%)	
NSVT	1 (1%)	
Prior Closed Mitral commissurotomy (CMC)	5(4.8%)	
Prior PTMC	11(10.6%)	
Prior CVA (Ischemic/Hemorrhagic stroke/TIA)	7 (6.3%)	
**LVEF**		
Normal LVEF	92 (88.46%)	
Mild LVSD	5 (4.8%)	
Moderate LVSD	5 (4.8%)	
Severe LVSD	2(1.92%)	
Pregnant	5(4.8%)	
**Mitral valve area**		
MVA pre PTMC [Mean ± SD]	0.983 ± 0.191	<0.01*
MVA post PTMC [Mean ± SD]	1.690 ± 0.198	
**Wilkin's score prior to PTMC**		
≤8	1.67 ± 0.20	0.003
>8	1.81 ± 0.15	

*Statistically significant.

**Fig. 1:**
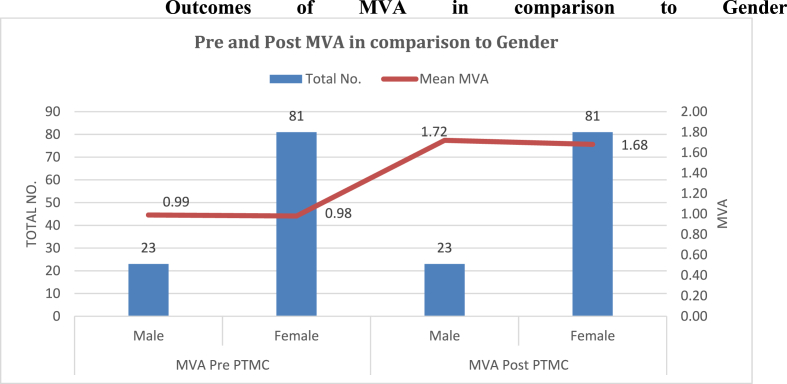
**Outcomes of MVA in comparison to Gender**.

Mean mitral valve area prior to PTMC was 0.98 ± 0.19 mm^2^ that increased to 1.69 ± 0.19 mm^2^ after the procedure. The increase in MVA was found to be statistically significant (p=<0.001) (Fig. 1). We found that post PTMC MVA varied with PTMC Wilkin's score with less than or equal to 8 having favorable outcomes (Table 2).

**Table 2:** Comparison of outcomes of pre and post PTMC.

	Pre PTMC	Post PTMC	P value
Mitral valve area	1.98 ± 0.19	1.69 ± 0.19	<0.001
Systolic LA Pressure	39.95 ± 10.57	23.96 ± 7.87	
Diastolic LA pressure	18.52 ± 6.72	8.88 ± 4.57	
Diastolic gradient	13.08 ± 4.18	5.74 ± 2.35	
PASP	48.98 ± 10.21	36.28 ± 9.99	
MR severity			
None	18 (17.3%)	5 (4.8%)	<0.001
Trivial	45 (43.3%)	10 (9.6%)	
Mild	39 (37.5%)	67 (64.4%)	
Moderate	2 (1.9%)	19 (18.3%)	
Severe	0 (0%)	3 (2.9%)	

Our study showed an overall success rate of 89.4%. In pregnant women, the mean gestational age at the time of procedure was 25.2 ± 4.5 years. Out of 5 pregnant women who underwent PTMC, it was successful in all the patients as the MVA increased from 0.98 ± 0.15 to 1.63 ± 0.25 cm^2^ with significant reduction of Mean LA pressure.

Local complications like hematoma formation in the puncture site occurred in 7(6.7%) of the patients. Femoral AV pseudoaneurysm was noted in 2 (1.92%) of the patients due to inadvertent puncture. Coronary embolization was seen in 1(0.96%) of the patients who developed acute Anterolateral ST Elevation Myocardial Infarction during the procedure itself. Hemopericardium was noted in 2(1.92%) of the patients. The details of other various complications are included in Table 3.

**Table 3:** Complications in patient that underwent PTMC.

Local Vascular Access site related	Hematoma/oozing	7
	Femoral pseudoaneurysm	2
Cardiac	Severe Mitral Regurgitation	3
	Pericardial effusion/Hemopericardium	2
	Ventricular arrhythmias: Non sustained VT	3
	Sustained VT	1
	AV Block – 2:1	2
	Left ventricular perforation	0
	Cardiac Tamponade	1
	Need of MVR	2
	Myocardial infarction	1
Embolic events	CVA (Ischemic/Hemorrhagic)	1
	Coronary Embolization	1
**Death**		0

## 5 Discussion

In this study, we found that there was increase in mean mitral valve area and was statistically significant. The overall success rate after the procedure was 89.4%. There was no reported death among the patients and hematoma formation was most common complications.

In our study, most common age group of the patients was 30–50 years; which is in accordance to the natural history of disease and various epidemiological studies. Female preponderance was noted in a high proportion of the patients at all ages; well supported by the available literatures [9]. Older age, a smaller valve area, a prior commissurotomy, or baseline mitral regurgitation are all to be taken into consideration as potential indicators for a worse immediate outcome with a predictive strength comparable to valve calcification, regardless of the echo grading method that is employed [10]. The hemodynamic profile of the patients changes with age. The elderly patients have a more severely calcific mitral stenosis and the young patients (<50 years old) with favorable valve morphology have typically displayed very strong immediate outcomes in clinical practice. In our study, pre PTMC valve area was found to be smaller in elderly (0.89 ± 0.19 cm^2^) than in younger age group (1.03 ± 0.16 cm^2^), which was statistically significant. The younger individuals showed a greater hemodynamic improvement after balloon dilatation, reflecting the lesser degenerative changes in the mitral valve. Atrial fibrillation is common in MS; more so in elderly and occurs in around 40% cases. Most of the patients (66.6%) who had atrial fibrillation were of age more than 60 years.

Our results of an 89.4% success rate for PTMC are in line with several other published research ranging from 80 to 96% [[11], [12], [13]]. The successful PTMC in pregnant female patients were in accordance to the study done by Esteves et al. [14] who observed the best short- and long-term outcomes in pregnant patients who received PBMV in the second trimester of their pregnancies. The increment in mitral valve area post procedure showed that the intervention was successful in patients with a pre procedural mitral valve area Wilkin's score of less than 8 than that of more than or equal to 8. These results were in line with a prior study done by Palacios et al. [15] which shown a significant positive influence on the immediate after PTMC in patients with a Wilkins score under 8. However, a positive outcome could also be attained in cases with a high score, according to several additional researchers who have discovered that mitral valve anatomy was the best predictor of mitral opening. The fallacies of the Wilkin's score lies in that it lacks two vital parameters namely commissural calcification and the degree of MR.

The end point of immediate procedural success is most often a final valve area more than 1.50 cm^2^ without moderate or severe mitral regurgitation. In most of the successful cases post PBMV, mitral valve area approximately doubles. This usually occurs in 80%–90% of cases and leads to an immediate symptomatic relief with approximately 50%–60% decrease in trans mitral gradient. In our study, there was a statistically significant increase in final valve area from 0.98 ± 0.19 cm^2^ to 1.6903 ± 0.19 cm^2^ without significant mitral regurgitation in most of the cases. Also, a significant decrease in mean trans mitral diastolic gradient was noted from 13.08 ± 4.18 to 5.74 ± 2.35. Similarly, a decline in mean LA pressure was noted from 25.84 ± 7.52 to 14 ± 5.16 mm Hg which is in accordance with various available literatures [[16], [17], [18]]. In our study, 2 patients had LAA clot despite which they underwent the procedure, a clot confined to LA appendage are relative contraindication only. Significant mitral regurgitation in the form of moderate to severe mitral regurgitation occurred in 19 (18.3%) and 3 (2.9%) of total patients, which is similar to other studies in which the severe mitral regurgitation varies from 1.4% to 9.4% [15,16,19]. Very infrequently, mitral regurgitation necessitates emergency surgery, noted to be around 0.3–3.3% of cases in a study by J K Harrison et al. [20] Elective Surgical MVR was done in two of our patients. In particular in elderly patients with significant stenosis, the stepwise inflations necessitate close monitoring of changes in the left atrial pressure and waveform to identify the increase in severity of MR. Patients may be at risk of developing moderate or severe MR following additional balloon dilations if they do not experience a significant decrease in left atrial mean pressure and v wave pressure during stepwise inflations of the balloon.

The low incidence of severe MR could thus be explained by the meticulous and careful serial stepwise balloon dilatation of the stenotic valve. Also, In cases where substantial commissural tear is the underlying cause, the severity of MR tends to lessen over time [21]. Thus, the complete echocardiographic evaluation of the mitral valve is of utmost importance to prevent severe MR. Favorable outcomes were not seen with Previous valvotomy cases that included previous closed mitral commissurotomy - 5 (4.8%) and PTMC - 11(10.6%) as those valves were less pliable and had higher commissural calcification and were thus less amenable to balloon valvotomy.

Local complications like hematoma formation in the puncture site occurred in 8(7.69%) patients related to high and low femoral puncture. Femoral AV pseudoaneurysm was noted in 2(1.9%) patients due to inadvertent puncture. Coronary embolization was seen in one of the patients who developed Acute ST Elevation Myocardial Infarction during the procedure itself. One of the most common indications for emergency surgery is hemopericardium, which occurs during BMV at a rate of between 0.6 and 4%. [22,23], was noted in 2 of our patients. In patients with favorable valve morphology, emergency surgery for hemopericardium should only be performed in cases of persistent collection after BMV [24]. Also, in the same study, 84.6% of patients who underwent PTMC with a second septal puncture had successful outcomes, eliminating the need for surgery. In our case, the pericardial effusion resolved later as evidenced by the reduction in effusion on serial echocardiographic monitoring. Cardiac tamponade occurred in one patient requiring emergent surgical clot evacuation, probably related to the high puncture of IAS because as the free wall of LA enlarges, IAS is pushed inferiorly as mentioned in a study by Varma et al. [23] It has been postulated that sites where puncture can occur are LA roof (if the septal puncture is high and the movement of sheath over IAS plane is not smooth), LA free wall (due to manipulation of guide wire), the right atrial side of IAS often overlies the Left Atrial side of IAS so that the needle may enter the pericardial space before going into LA, LV injury (due to the over the wire balloon technique with a guide wire tip), Posterior RA wall tear (due to low septal puncture) or sometimes even pulmonary vein inflation with the balloon. With the invention of pigtail-tip balloon catheters and the application of septal puncture with correct identification and catheter landmarking of the aortas in right anterior oblique view, the risk of aortic rupture is low [25]. In one of the patients, coronary embolization leading to Acute ST Elevation MI was observed, which was recognized promptly and managed with intensive ICU monitoring, judicious use of antiplatelets and anticoagulants. This rare occurrence has also been described in a study by Kadiyala et al. [26].

This study was the first of its kind from our center to assess the clinical profile and outcomes of the patient undergoing PTMC. Our study had several limitations. First, only one data source in the center was included in the sample size. The single-center design of the study may prevent it from being applicable to the entire population of Nepal. However, given that morbidity rates in practice do not significantly differ across the nation, and considering that our hospital serves as a referral center for Nepal, the patient pool should be considered to be sizeable and representative. Second, it is a retrospective study as the records did not have all the data. The results are in line with those of other studies and should help with the gathering of information on this subject. Furthermore, the cross-sectional design of the study makes it impossible to pinpoint the date of the diagnosis and its effects.

## 6 Conclusion

Meticulous assessment of pre-procedural echocardiographic mitral valve parameters appears to be helpful in predicting the favorable PTMC results. Successful PTMC is highly influenced by the patients' increasing age, valve morphology (calcification, thickness, mobility), Left atrial dimensions, pre-PTMC mitral valve area, degree of baseline MR. Post procedure development of MR is usually well tolerated but rarely be severe enough requiring surgical valve replacement.

## Provenance and peer review

Not commissioned, externally peer-reviewed.

## Sources of funding

No funding was received for the study.

## Ethical approval

Ethical approval was taken from Institutional Review Committee of Institute of medicine.

## Consent

Written informed consent was obtained from the participant for participation in this study.

## Author contribution

SY and SS wrote the original manuscript, reviewed, and edited the original manuscript. SY, SS, RMG, CMP, RG, and NS reviewed and edited the original manuscript.

## Registration of research studies

1. Name of the registry: Research registry
2. Unique Identifying number or registration ID: researchregistry8150
3. Hyperlink to your specific registration (must be publicly accessible and will be checked): Register Now - Research Registry

## Guarantor

Dr Sutap Yadav.

## Declaration of competing interest

Authors have no conflict of interest to declare.
